# Wearable Proximity Sensors for Monitoring a Mass Casualty Incident Exercise: Feasibility Study

**DOI:** 10.2196/12251

**Published:** 2019-04-26

**Authors:** Laura Ozella, Laetitia Gauvin, Luca Carenzo, Marco Quaggiotto, Pier Luigi Ingrassia, Michele Tizzoni, André Panisson, Davide Colombo, Anna Sapienza, Kyriaki Kalimeri, Francesco Della Corte, Ciro Cattuto

**Affiliations:** 1 Data Science Laboratory Institute for Scientific Interchange Foundation Torino Italy; 2 Department of Translational Medicine Eastern Piedmont University Novara Italy; 3 Centro Interdipartimentale di Didattica Innovativa e di Simulazione in Medicina e Professioni Sanitarie SIMNOVA Università del Piemonte Orientale Novara Italy; 4 Department of Design Politecnico di Milano Milano Italy; 5 University of Southern California Information Sciences Institute Marina del Rey, CA United States

**Keywords:** contact patterns, contact networks, wearable proximity sensors, mass casualty incident, simulation, medical staff – patient interaction, patients’ flow

## Abstract

**Background:**

Over the past several decades, naturally occurring and man-made mass casualty incidents (MCIs) have increased in frequency and number worldwide. To test the impact of such events on medical resources, simulations can provide a safe, controlled setting while replicating the chaotic environment typical of an actual disaster. A standardized method to collect and analyze data from mass casualty exercises is needed to assess preparedness and performance of the health care staff involved.

**Objective:**

In this study, we aimed to assess the feasibility of using wearable proximity sensors to measure proximity events during an MCI simulation. In the first instance, our objective was to demonstrate how proximity sensors can collect spatial and temporal information about the interactions between medical staff and patients during an MCI exercise in a quasi-autonomous way. In addition, we assessed how the deployment of this technology could help improve future simulations by analyzing the flow of patients in the hospital.

**Methods:**

Data were obtained and collected through the deployment of wearable proximity sensors during an MCI functional exercise. The scenario included 2 areas: the accident site and the Advanced Medical Post, and the exercise lasted 3 hours. A total of 238 participants were involved in the exercise and classified in categories according to their role: 14 medical doctors, 16 nurses, 134 victims, 47 Emergency Medical Services staff members, and 27 health care assistants and other hospital support staff. Each victim was assigned a score related to the severity of his/her injury. Each participant wore a proximity sensor, and in addition, 30 fixed devices were placed in the field hospital.

**Results:**

The contact networks show a heterogeneous distribution of the cumulative time spent in proximity by the participants. We obtained contact matrices based on the cumulative time spent in proximity between the victims and rescuers. Our results showed that the time spent in proximity by the health care teams with the victims is related to the severity of the patient’s injury. The analysis of patients’ flow showed that the presence of patients in the rooms of the hospital is consistent with the triage code and diagnosis, and no obvious bottlenecks were found.

**Conclusions:**

Our study shows the feasibility of the use of wearable sensors for tracking close contacts among individuals during an MCI simulation. It represents, to our knowledge, the first example of unsupervised data collection—ie, without the need for the involvement of observers, which could compromise the realism of the exercise—of face-to-face contacts during an MCI exercise. Moreover, by permitting detailed data collection about the simulation, such as data related to the flow of patients in the hospital, such deployment provides highly relevant input for the improvement of MCI resource allocation and management.

## Introduction

### Background

A mass casualty incident (MCI) is defined as a situation in which, at a certain time, the available care resources are unable to meet the demand for medical care of the incident [1]. Each year, MCIs occur worldwide and are caused by conventional causes such as weapons, explosions, vehicular and airplane accidents, and deliberate or spontaneous chemical mass intoxications. These incidents require emergency health care teams to treat large numbers of injured victims [2], and this might compromise the normal functioning of hospitals. Simulation applied to health care is rapidly gaining acceptance in medical and academic communities, and it can be a valuable tool for better training for management of MCIs. It provides a safe, controlled environment in which it is possible to test plans and procedures and improve them, as well as to evaluate policies and guidelines [3]. Traditionally, actors are used in disaster exercises, and they are coached to mimic and exhibit realistic manifestations of several medical and traumatic pathologic states that may be present in a real MCI [2-4]. Although the use of simulation in medical education has increased over the last 2 decades, collecting and analyzing data of a mass casualty functional exercise still occurs in an unsystematic manner, without a standardized method. Common methods to assess performances during MCI simulations are direct observations of functional exercise performance and video analysis of participants’ behaviors [5]. However, these methods present some limitations: the focus of the observers’ attention can subjectively vary, and the need of several observers, both for direct observations and for videos, could affect the realism of the event and decrease the level of emotional engagement of the participants [3]. An objective and reproducible method that identifies the strengths and weaknesses of simulations is required to lead the improvement in the response system [6]. In mass casualty simulations, wireless medical sensor networks and Radio-Frequency IDentification (RFID) technology have been used to track information about the status of the casualties, thus providing timely situational awareness during exercises [3,7,8], and the use of RFID was compared with manual data collection, demonstrating the reliability and applicability of the system [3]. Wearable proximity devices could provide not only patient information and tracking capability to locate people and equipment but also information on interactions among individuals. Wearable sensors have been successfully used to measure face-to-face proximity relations in various hospital settings that include the pediatric ward [9] and acute care geriatric unit [10,11].

### Objectives

The use of proximity sensors in the field of MCI simulation could provide a continuous and fully distributed collection system of high-resolution data on the interactions among patients and medical staff to investigate the dynamics of interactions with regard to the different roles and severity of the patient’s injuries. In this study, we have illustrated the feasibility of contact measures through wearable proximity sensors in a live MCI simulation aimed at providing data-driven knowledge to perform debriefing and identify room for improvement. The main objectives of our study were (1) to investigate the interactions between medical staff and patients with regard to their roles and severity of the victim’s condition and (2) to estimate the presence of victims in different spaces of the field hospital to study the patients’ flow.

## Methods

### Study Setting

A building collapse following a flood was simulated during an MCI functional exercise organized in Novara, Italy, on May 19, 2016, from 7 pm to 10:30 pm. The MCI exercise was organized in the framework of the residential course of the European Master in Disaster Medicine (EMDM). The EMDM is an international 12-month–long blended learning master’s degree program for health care providers involved in medical preparedness and response to disasters [12]. The exercise included both a prehospital and an in-hospital disaster response phase. The scenario comprised 2 locations: the building collapse site (prehospital response) and the field hospital (in-hospital response). The hospital was located approximately 2 kilometers from the incident site.

Overall, exercise participants were distinguished into the following classes for the purpose of this study and based on their role in the simulation: Medical Doctors (*MD*), Nurses (*Nurse*), Emergency Medical Service (*EMS*) personnel, and Health Care Assistants (*HCA*) and simulated victims (*Victim*). Victims were portrayed by medical students. They attended an introductory course on disaster medicine (8-hour live lectures) and specific training on how to simulate clinical conditions provided in an individual victim storyboard, when to change the dynamic casualty cards (DCCs) reporting their vitals according to the treatment applied, and how to properly collect data (2-hour live lectures). Details about the casualty evolution method, general structure of the simulation and DCCs were described in a series of previous papers [3,6,13,14]. The EMDM students acted as doctors and nurses and were distributed as follows: 8 physicians and 5 nurses staffed the ambulances provided by local EMS agencies as prehospital response, whereas 6 physicians and 11 nurses were in the field hospital that had been previously deployed by the Italian Army. EMS personnel and HCAs were played by local ambulance volunteers (basic emergency medical technician level) and first aid–trained soldiers, respectively. None of the participants had been previously informed about the scenario.

### Expected Triage and Injury Severity Score

According to their predetermined storyboard, each victim had an expected initial triage category according to the Simple Triage and Rapid Treatment protocol [15]: 6 victims were *Black*, 15 were *Red*, 27 were *Yellow*, and 86 were *Green*. Responders had to assign a triage score to each victim during the exercise, both at the accident site (*on-scene triage*) and at the hospital (*hospital triage*). In Section 2 of the Multimedia Appendix 1, we report the final triage accuracy of the exercise.

Victims (*Black* group excluded) were also classified based on their injuries using the Injury Severity Score (ISS) [16]. The ISS is an established medical score to assess trauma severity with a range from 1 to 75, grouped by 5 categories: *Minor* (1-3), *Moderate* (4-8), *Serious* (9-15), *Severe* (16-24), and *Critical* (25-75). In addition, the category *Nontraumatic* that indicates victims without physical trauma (such as anxiety crises) was added. In total, 21 victims were assigned to the *Nontraumatic* group, 36 to *Minor* group, 47 to *Moderate* group, 9 to *Serious* group, 4 to *Severe* group, and 11 to *Critical* group.

### Data Collection

**Figure 1:**
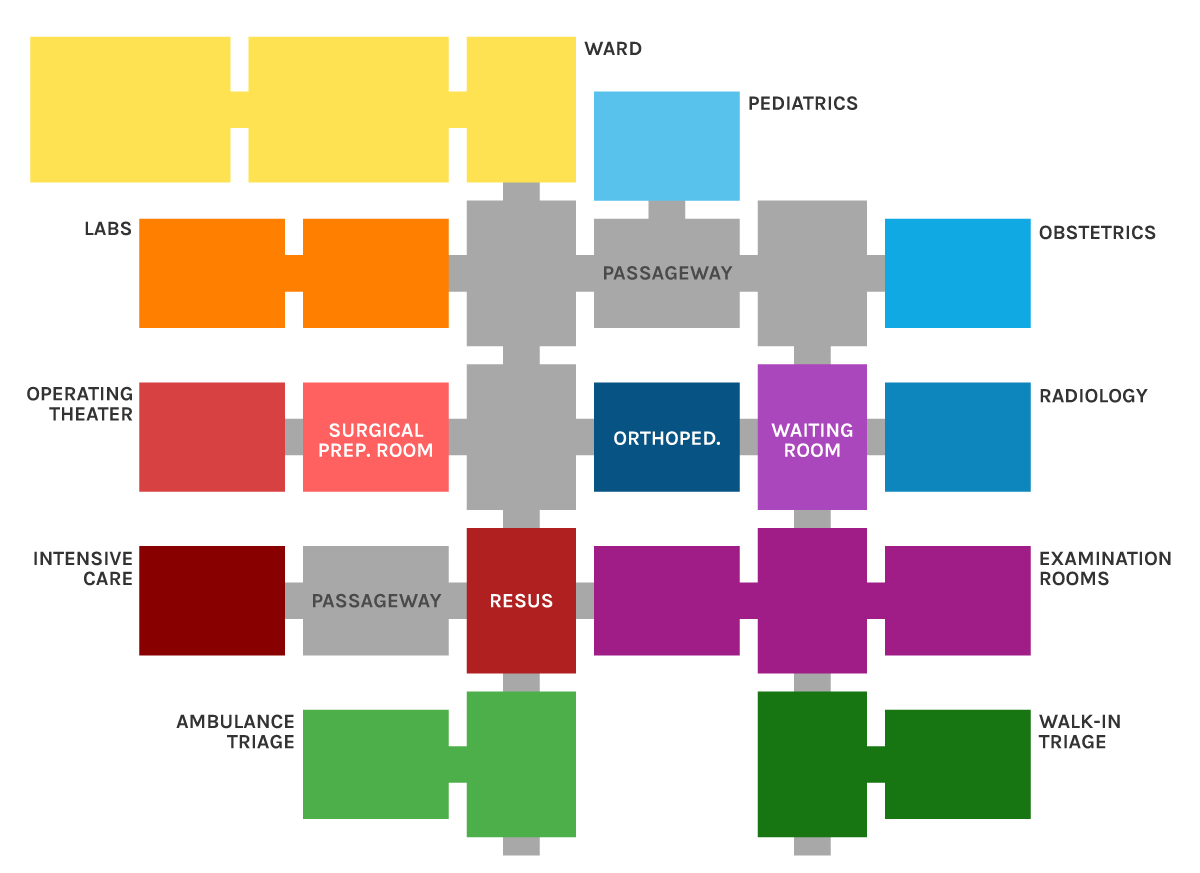
A map of the field hospital.

Data collection was performed as described below. Each participant wore a wearable proximity sensor: the sensor was inserted into a transparent envelope and fixed with adhesive tape at the center of the chest (on the sternum area) to detect person-to-person interactions. At the beginning of the simulation, victims were both at the accident site and at the hospital as regular in-hospital patients. During the exercise, victims could take 1 of 3 possible pathways: (1) transferred from the accident sites to the hospital by ambulances and minibuses; (2) transferred to another virtual hospital; and (3) discharged from the simulation. The exact times of the transfers as well as the ending time of the simulation for each victim were marked by external observers. In addition, proximity sensors were placed on the ceiling of the rooms (tents) of the hospital area (Figure 1; category *Location*) as fixed tags. Table 1 reports a summary of the total number of sensors for each category in the Prehospital and Hospital area.

The sensor setup was designed by the SocioPatterns collaboration consortium [17]. This system is based on wearable proximity sensors (*tags*) that exchange ultra–low power radio packets in a peer-to-peer fashion [9,18-20]. Sensors in close proximity exchange with one another a maximum of about 1 power packet per second, and the exchange of low-power radio packets is used as a proxy for the spatial proximity between tags [9,18]. In particular, close proximity is measured by the attenuation, defined as the difference between the received and transmitted power. Each device has a unique identification number that was used to link the information on the individual carrying the device with his/her profile or, in the case of fixed tags, with the location where the sensors are placed. More details on the functioning of the tags and on the data collection pipeline are given in Section 1 of the Multimedia Appendix 1.

**Table 1:** Number of proximity sensors in the Prehospital and Hospital area by category.

Category	Prehospital	Hospital	Total
Medical Doctor	8	6	14
Nurse	5	11	16
Victim	112	22	134
Emergency Medical Service	47	—^a^	47
Health Care Assistant	—	27	27
Location	—	30	30
Total	172	96	268

^a^Not applicable.

### Contacts Among Participants

We analyzed the contacts among participants belonging to categories *Victim*, *MD*, *Nurse*, and *EMS* for the Prehospital area and the contacts between participants belonging to categories *Victim*, *MD*, *Nurse*, and *HCA* for the Hospital area. We considered the contacts between individuals across categories, both in the Prehospital and Hospital area. We defined that a *contact* occurs between 2 individuals during a time slice duration of 20 seconds if the proximity devices worn by the participants exchanged at least 1 radio packet during that interval and the median attenuation of received packets exceeds an attenuation threshold of 70 dBm. After a contact is established, it is considered ongoing as long as the devices continue to exchange at least 1 such packet for every subsequent 20-second interval [18]. The system was set to detect proximity events between devices situated within 1 to 1.5 m of one another. This setting ensures that when individuals wear the devices on their chest, exchange of radio packets between devices is only possible when they are facing each other, as the human body acts as a radio-frequency shield at the carrier frequency used for communication. This system allows us to monitor the number of contacts and their duration. Data were extracted and cleaned separately for each sensor, and those collected before 7 pm and after 10:30 pm were discarded to keep track of only meaningful proximity events. Moreover, for each victim, we discarded the data collected after the exact time of simulation end (death, discharge, or end-of-simulation time). We analyzed the data separately for the Prehospital and Hospital area. With regard to the victims transferred from the accident site to Hospital during the simulation, we considered the data collected before the exact time of transfer belonging to Prehospital data and data collected after this time belonging to Hospital data.

We generated aggregated networks of contacts between participants on the full exercise duration, both in the Prehospital and Hospital area, to study the close range interactions during the exercise as well as to confront the results with those obtained in different real-world settings. We call *k*_*i*_ the degree of a node *i*, ie, the number of distinct individuals with whom individual *i* has been in contact, and *w*_*ij*_, the weight of an edge between nodes *i* and *j,* ie, the cumulative duration of the contact events recorded between 2 individuals, *i* and *j*.

Then, we generated contact matrices based on the median cumulative time spent in proximity between victims with different triage and ISSs and the caregivers (medical doctors, nurses, emergency medical services, and health care assistants). Time spent in proximity with victims for each caregivers’ category was compared using the Kruskal-Wallis test. We respectively considered the *on-scene triage* scores and the *hospital triage* scores to build the Prehospital and Hospital matrix (ie, the triage scores assigned by the medical doctors).

### Presence of Victims in the Field Hospital

We estimated the presence of the patients in different rooms of the field hospital by analyzing the power packets exchanged between sensors worn by individuals belonging to the category *Victim* and the fixed sensors belonging to category *Location* in the Hospital area. To assess the location of a patient in a given room at a given time, we set up 2 thresholds on the count of power packets exchanged between the devices respectively to evaluate the presence of the participant in the exercise and the presence of the participant in a given room. For each time slot of 5 min, we assumed that a participant is still present in the exercise site if the total count of the power packets exchanged between all the fixed tags and the participant’s tag is greater than 15. We assumed that a participant is present in a given room if the total number of power packets exchanged between his/her tag and the fixed tag of the room considered is higher than 5 for each time slot.

This allows describing the patients’ flow through the rooms of the field hospital. The field hospital consisted of 27 rooms organized as follows: 3 general wards, 2 laboratories, 5 passageways (hallways), 3 examination rooms, 1 pediatric ward, 1 waiting room, 1 obstetric ward, 1 operating theatre with 2 beds, 1 surgical preparation room, 1 orthopedic ward, 1 radiology waiting room, 1 radiology, 1 intensive care with 3 beds, 1 emergency department resuscitation (resus) area, 2 ambulance triage rooms (patients brought in by ambulance), and 2 walk-in triage rooms. In this analysis, we grouped the patients’ wards, the laboratories, the passageways, the examination rooms, the ambulance triage rooms, and walk-in triage rooms in the same space.

### Presence Patterns of Victims in the Hospital

To study the link between the presence patterns of victims and the conditions of the victims, we used the t-Stochastic Neighbor Embedding (t-SNE) technique that converts a high-dimensional data set into a matrix of pair-wise similarities and allows to visualize the resulting similarity data [21]. In this study, we used, as an input dataset, a set of vectors where each vector described the spatial features of each victim. More exactly, each victim is initially represented as a vector where elements are time spent by that patient in a given room of the field hospital (normalized on total presence duration).

## Results

### Network Analysis and Contact Among Victims and Rescuers

**Figure 2:**
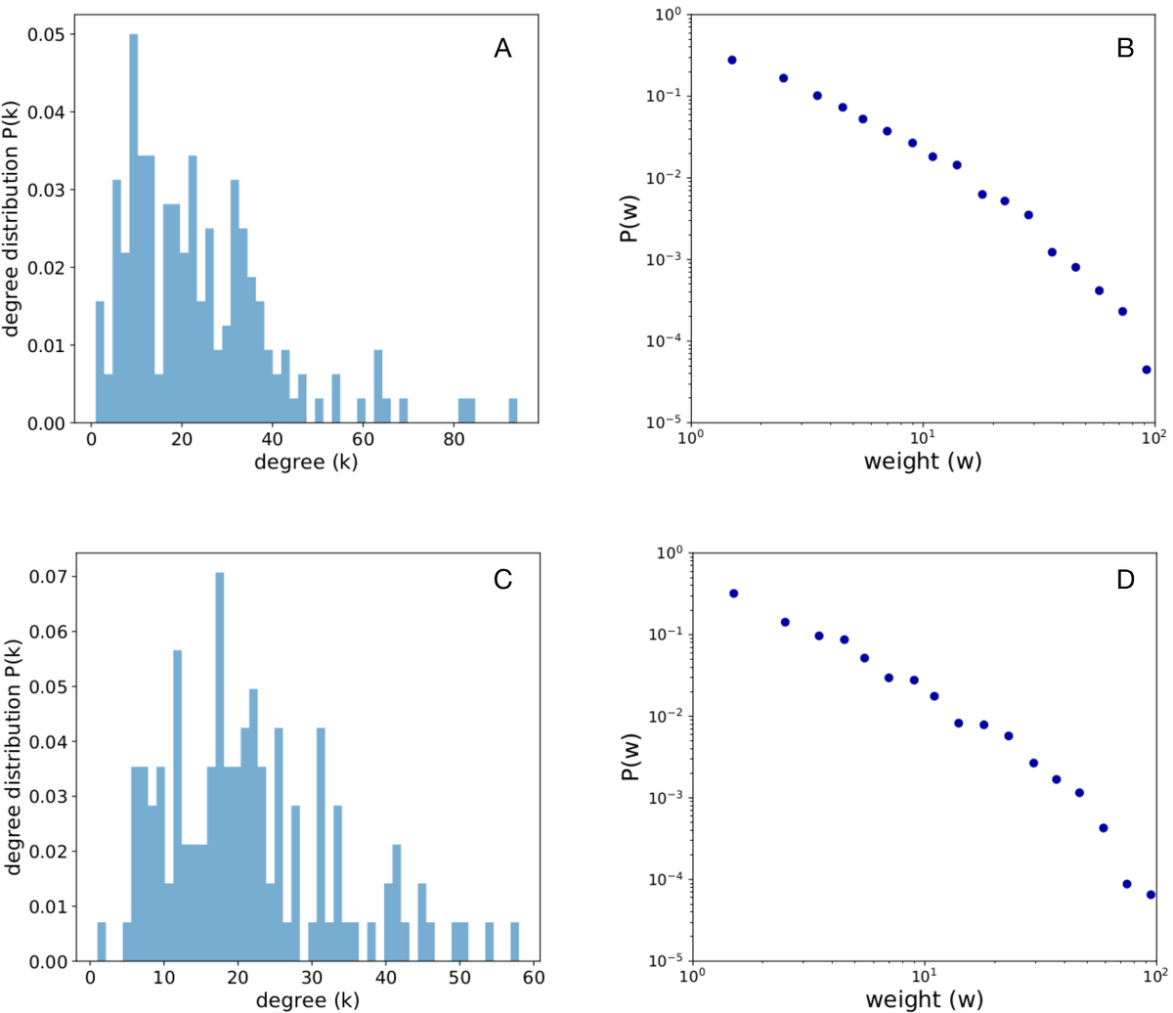
Degree and weight distributions. Degree distribution P(k) of the aggregated contact networks, in the Prehospital area (panel A) and in the Hospital area (panel C). Distribution of the weights of the aggregated contact networks in the Prehospital area (panel B) and in the Hospital area (panel D).

A total of 238 individuals participated in the exercise. They were categorized as follows: 14 MD, 16 Nurses, 134 Victims, 47 EMS, and 27 HCA. The contacts within the same category were not included in this analysis. Figure 2 shows the degree and weight distribution in the Prehospital and Hospital area. The aggregated contact network in the Prehospital area is formed by 172 nodes and 2035 edges, and the average degree is k=23.66 (range 1-94), and in the Hospital area, the network is formed by 124 nodes and 1335 edges, and the average degree is k=21.53 (range 1-58). The weight distribution is heterogeneous in both areas, with heavy-tailed distributions: most contacts are short, and there are few long-lasting contacts.

**Figure 3:**
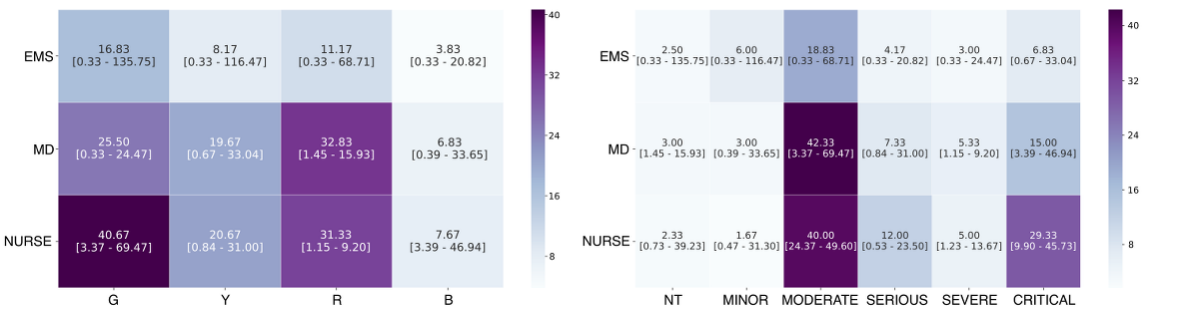
Prehospital contact matrices. Median of cumulative time spent (in minutes) between patients with different triage score and rescuers (left panel); Median of cumulative time spent (in minutes) between patients with different Injury Severity Scores (Nontraumatic, Minor, Moderate, Serious, Severe, and Critical) and rescuers (right panel). 95% CIs are indicated in brackets. EMS: Emergency Medical Services; MD: Medical Doctors; NT: nontraumatic; G: Green; Y: Yellow; R: Red; B: Black.

**Figure 4:**
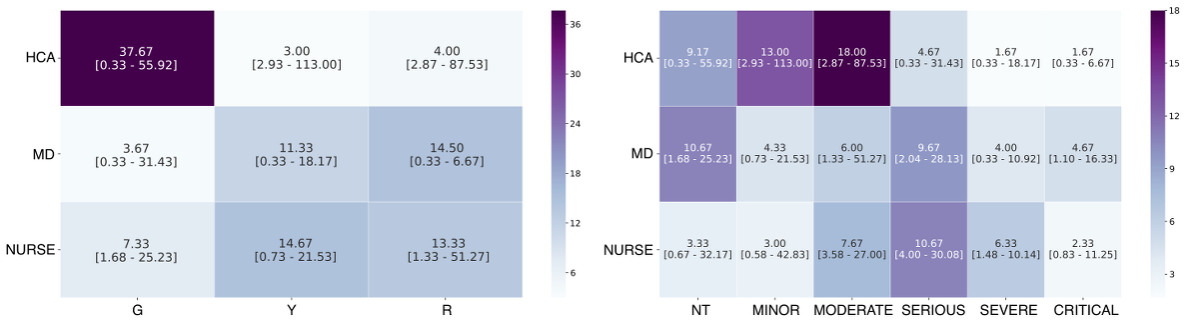
Hospital contact matrices. Median of cumulative time spent (in minutes) between patients with different triage score and rescuers (left panel); Median of cumulative time spent (in minutes) between patients with different Injury Severity Scores (Nontraumatic, Minor, Moderate, Serious, Severe, and Critical) and rescuers (right panel). 95% CIs are indicated in brackets. HCA: Health Care Assistants; MD: Medical Doctors; NT: nontraumatic; G: Green; Y: Yellow; R: Red.

Contact matrices reveal different amounts of time spent in proximity depending on the severity of the patient and the role of the caregiver (Figures 3 and 4). At the scene of the accident, there was a significant difference between the time spent in proximity between EMS and victims both with regard to the triage (X^2^_3_=19.479; *P*<.001) and to the ISS (X^2^_5_=36.106; *P*<.001). The higher time in contact was with Green victims and with victims classified as Moderate. Regarding the triage, there were no significant differences between the time spent in proximity with the victims for both MD and nurses. On the contrary, regarding the ISS, there were significant differences for MD (X^2^_5_=13.576; *P*=.02) and nurses (X^2^_5_=12.798; *P*=.02). Both categories spent higher time in contact with victims classified as Moderate and Critical. At the field hospital, there was a significant difference between the time spent in proximity between HCA and victims both with regard to the triage (X^2^_3_=31.271; *P*<.001) and to the ISS (X^2^_5_=46.989; *P*<.001). The higher time in contact was with Green victims and with victims classified as Minor and Moderate. There were no significant differences between the time spent in proximity between MD and victims (with regard to the triage and ISS) and nurses and victims (with regard to the triage). However, there was a significant difference between the time spent in proximity between nurses and victims with regard to the ISS (X^2^_5_=14.965; *P*=.01), nurses spent the higher time in contact with victims classified as Moderate and Serious.

**Figure 5:**
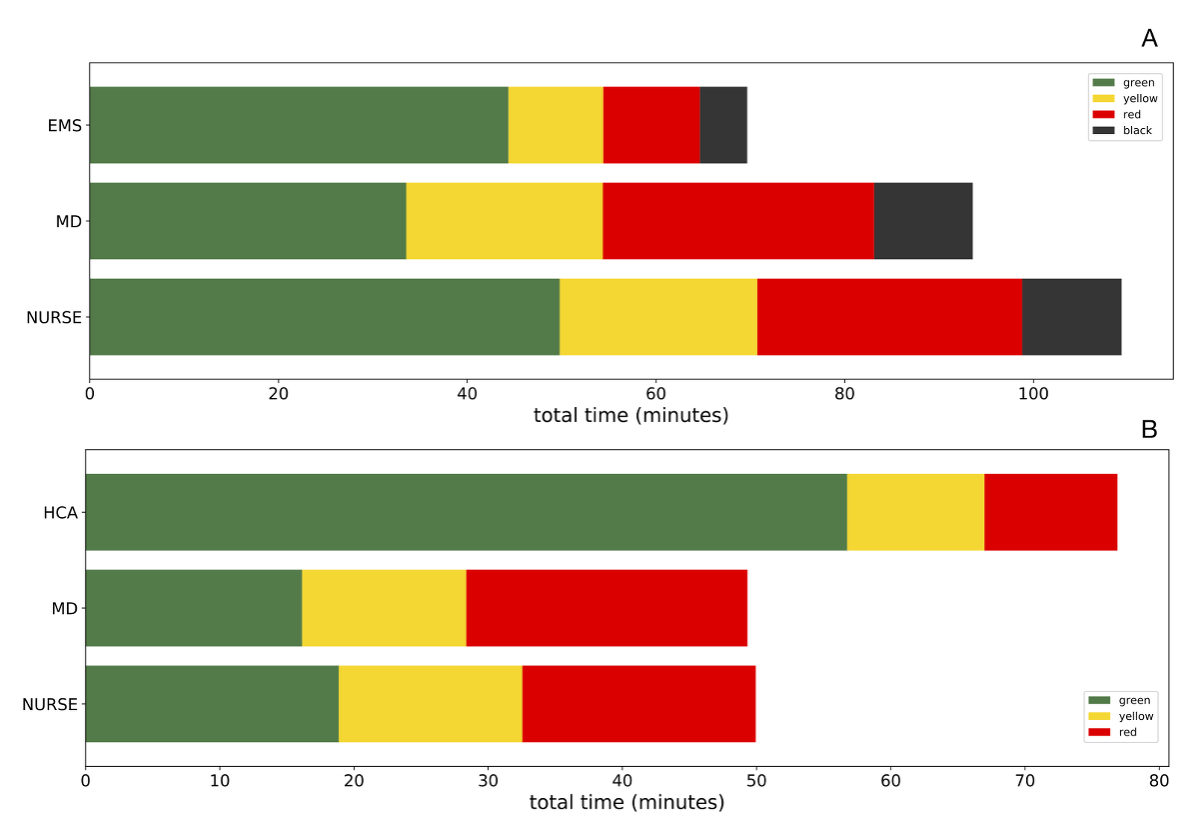
Cumulative time in contact (normalized on the total number of participants belonging to each caregiver category) between caregivers and victims with different triage at the Prehospital area (panel A) and Hospital area (panel B). EMS: Emergency Medical Services; HCA: Health Care Assistants; MD: Medical Doctors.

Figure 5 shows the cumulative time in contact (normalized on total number of participants belonging to each caregiver category) between caregivers and victims with different triage at the Prehospital area (panel A) and Hospital area (panel B). The rescuers who spent more cumulative time in contact with victims were nurses at the scene of the accident and HCA at the field hospital.

### Casualty Flows in the Hospital

**Figure 6:**
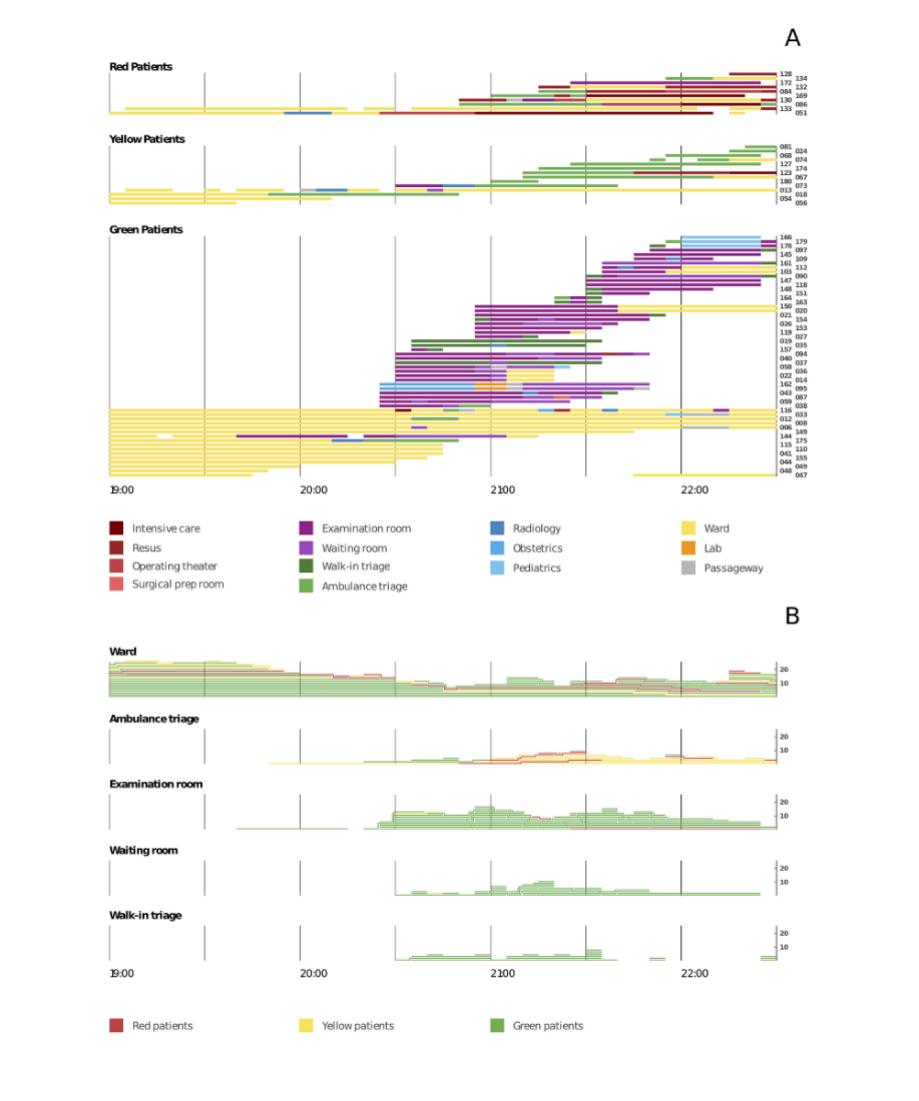
Panel A: Victims flow through the field hospital. Each bar represents a patient (code is indicated on the right of the bar), the color of the bar’s segments refers to the room of the field hospital, and the length of the segments represents time spent by the victim in each room. Numbers on the right part of the figures are the identification number for each victim. Panel B: Number of victims in the Ward, Ambulance triage, Examination room, Waiting room, and Walk-in triage over the simulation period. Each line corresponds to the presence of a victim; the color corresponds to the triage code. Numbers on the right part of the figure indicate the number of people in the room.

We studied the flow of 80 patients: 22 victims were already in the field hospital at the start of simulation as regular in-hospital patients and 58 victims were transferred from the accident site (the first transfer occurred at 8:15 pm). Figure 6, panel A, shows the victims flow through the field hospital of 56 patients with Green triage, 14 patients with Yellow, and 10 patients with Red triage. Each bar represents a patient, the color of the bar’s segments refers to the room of the field hospital, and the length of the segments represents time passed by the victim in each room. The presence of patients in the different rooms of the hospital is consistent with triage code and diagnosis. Green victims passed most of their time in the Ward, Examination room, Walk-in triage room, and Waiting room. A total of 11 Yellow patients out of 14 spent time in the Ambulance triage, 6 Red victims out of 10 spent time in resus, and 3 Red victims spent time in the Intensive care. Figure 6, panel B, shows the number of victims in the Ward, Ambulance triage, Examination room, Waiting room, and Walk-in triage room over the simulation period. Each line corresponds to the presence of a victim; the color corresponds to the triage code.

By analyzing the flow, we aimed at detecting the potential presence of bottlenecks in the field hospital. To do this, we focused on the analysis of the presence of the victims in the rooms in which they were not receiving any medical treatments. We defined bottlenecks as situations where the time of victims spent in the rooms where they did not receive any treatment is increased compared with the average time normally observed (for instance, when the number of victims in the hospital is low). Such rooms in the present settings are as follows: Ambulance triage, Examination room, Waiting room, and Walk-in triage room. We compared the numbers of victims present at the same time at the same room in relation with the waiting time in Ambulance triage, Examination room, Waiting room, and Walk-in triage room; the Pearson correlation test was used. The mean waiting time in the Ambulance triage was 40 min (SD 9), in the Examination room was 40 min (SD 9), in the Waiting room was 39 min (SD 10), and in the Walk-in triage room was 31 min (SD 23). There were no significant correlations between the number of victims present in the same time at the same room and the waiting time; this result indicated that as the time passed in a room by a patient is not affected by the arrival of many victims in the hospital, in other terms, there was no obvious presence of bottlenecks.

**Figure 7:**
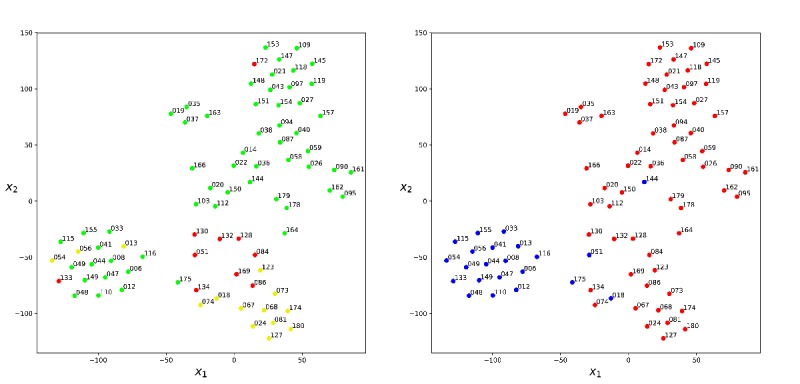
Presence patterns of individual victims in hospital rooms visualized using t-Distributed Stochastic Neighbor Embedding (t-SNE) to map high-dimensional patient vectors to a 2-dimensional feature space (*X_1_* and *X_2_* axes). Each point corresponds to a patient. Victims with similar presence vectors are mapped to neighboring points in the plane. Victims are color coded by triage code (left panel) and by the start location of the simulation (red: accident site; blue: field hospital).

We studied the presence patterns of individual victims in hospital rooms. For each patient we built a feature vector containing the time spent by that patient in each of the 15 hospital rooms normalized by total presence duration. The resulting set of 15-dimensional vectors (one per patient) was visualized using a dimensionality reduction technique known as t-SNE that maps each 15-dimensional patient vector to a 2-dimensional feature space (*X*_*1*_ and *X*_*2*_ axes; Figure 7). Clusters of patients with similar presence patterns are visible. We observed that the bottom-right cluster contains the more serious cases (yellow and red codes) and the top-right cluster contains the less serious cases (green codes), with the exception for the victim coded 172. The ideal triage code of the victim 172 was Yellow, and this victim passed the entire time of the simulation in the Examination room. On color coding by start location, the bottom-left cluster contains the majority of victims that started the simulation in the field hospital.

**Figure 8:**
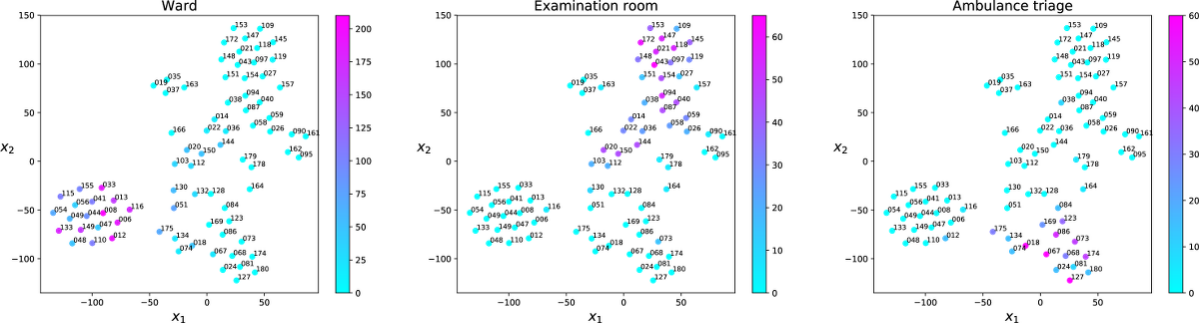
Presence patterns of individual victims in hospital rooms visualized using t-Distributed Stochastic Neighbor Embedding (t-SNE) to map high-dimensional patient vectors to a 2-dimensional feature space *X_1_* and *X_2_* axes). Each point corresponds to a patient. Victims with similar presence vectors are mapped to neighboring points in the plane. Points (patients) are color coded according to the time spent in minutes in the Ward (left panel), Examination room (middle panel), and Ambulance triage room (right panel).

Moreover, we studied the presence times of individual victims in 3 hospital rooms characterized by longer presence times of the victims (Figure 8). We observed that the bottom-left group of patients on the t-SNE plot are characterized by long presence times in the Ward and they correspond to the victims that started the simulation in the field hospital; the group of victims on the top of the plot are characterized by long presence times in the Examination room, and they correspond to the victims coded green, and the patients that spent more time in the Ambulance triage room are the more serious cases, coded yellow and red.

## Discussion

### Principal Findings

With this study, we report the first quantitative assessment of social contact patterns in live MCI simulation, based on wearable proximity sensors. Our study showed the feasibility of the use of wearable proximity sensors to measure contact patterns during an MCI functional exercise. We obtained simple charts and contact matrices which allow for direct visualization of potentially missed opportunities for improvement of the MCI response.

In this study, we used proximity sensors to evaluate the contacts between individuals and the flow of the victims during an MCI simulation. The system provides information about the simulation which is coherent with the severity of the diagnosis both from the point of view of the relations between caregivers and victims as well as of the flow of victims. Our results showed that there were no differences between the median time passed by medical doctors and nurses with victims with different triage, both in the Prehospital and Hospital area. At the scene of the accident, this result is consistent with the chaotic environment typical of a disaster, where the medical staff task allocation is challenging and the medical interventions are equally distributed between patients with different severity injuries. However, significant differences between time in proximity by medical doctors and nurses with victims with different ISSs were observed at the Prehospital area. Both caregiver categories spent more time in contact with patients with the most severe injuries (classified as Critical) and, in particular, with patients classified as Moderate in terms of the ISS. When presented and discussed critically at the debriefing, it turned out that the majority of Moderate victims suffered bone fractures, and owing to this, the immobilization and stabilization procedures required a lot of time before being mobilized and transferred to the field hospital. At the Hospital area, nurses spent higher time in contact with Moderate and Serious patients in terms of the ISS.

Although there are no significant differences between the time passed by medical doctors and nurses with victims with different triage, proximity sensors revealed that medical doctors and nurses spent relevant time with patients with Red and Yellow triage, both in the Prehospital and Hospital area. Minor wounded (green codes) were predominantly managed by the EMS staff on scene and HCA in the hospital, allowing medical doctors and nurses to spend more time with the patients in most need. The quantitative measurement of contact patterns provided the opportunity to debate about it during the debriefing and identifying further strategies and counter actions. In disaster and MCI environments, coordination for task allocation is challenging. The analysis of temporal features of contact links between caregivers and casualties revealed proportional resource utilization of different health care skills for different victim severity triages and ISS codes.

### Contact Data and Network Analysis

We obtained aggregated contact networks of the participants involved in the simulation, and we calculated the mean degree of the networks (ie, the mean number of connections between participants) for the whole duration of the simulation. Our results showed that the average degree was similar both for the in-Hospital and Prehospital area. The mean number of connections is lower for medical doctors and nurses than that of the EMS and HCA personnel. In other words, the medical staff interacted with a low number of patients by focusing treatment on a limited number of cases (see Section 3 of Multimedia Appendix 1). Moreover, the contact networks showed a high heterogeneity of the cumulative time spent in proximity (ie, weight of the edges) between participants, despite the short duration of the simulation. Our results show a highly heterogeneous distribution of contact durations characterized by a heavy tail; this outcome confirms the presence of a *universal* feature of contact patterns with most contacts of short duration and few long-lasting contacts. A similar general distribution of contact durations has been observed in other settings, including schools [20], hospitals [9], and households [22]. Moreover, the density of the networks (ie, fraction of all possible edges that are present in the network) was calculated to study the topology of networks built for each caregiver’s categories and victims. The networks are sparse in both scenarios, in particular for EMS and HCA. However, we found that the density varied through the severity of injuries of the victims for the medical doctors and nurses, which showed a higher number of potential connections with victims with more serious conditions (see Section 4 in the Multimedia Appendix 1).

The analysis of temporal evolution of the number of contacts between participants revealed a high concentration of contacts during the middle part of the simulation at the Prehospital area, from 8 pm to 9:30 pm, even after the transfer of a part of the victims to the field hospital. The peak at the end of simulation is most likely due to an artefact: the meeting of participants shortly before the collections of sensors. In the Hospital area, the number of contacts was stationary until the transfer of patients from the Prehospital to Hospital area, then the number gradually increased as expected (see Section 5 in the Multimedia Appendix 1).

### Flow and Presence Patterns of Victims in the Hospital

The deployment of sensors inside the hospital allowed to study the casualty flow. This analysis enabled to evaluate whether the patients were correctly headed by the health care personnel, consistently to the severity of the diagnosis and the expected location for such a diagnosis, in other words if patients with high acuity pathology were correctly occupying high acuity areas of the hospital and vice-versa, if low acuity patients were managed without wasting precious resources. Our results showed that the presence of patients in the hospital rooms were consistent with the triage code and diagnosis. Green victims spent most of their time in the examination room, Walk-in triage room, and Waiting room. A total of 6 Red victims out of 10 spent time in the Resus (ie, resuscitation area). A total of 3 Red patients spent most of their time in Intensive care (ie, Intensive Care Unit), and their diagnosis included head and chest trauma and septic shock. A total of 11 Yellow patients with minor injuries and stable trauma out of 14 spent time in the Ambulance triage. We studied the potential presence of bottlenecks for the rooms where victims were examined by medical staff, and they were waiting to receive treatments (Ambulance triage, Examination room, Waiting room, and Walk-in triage room) and evident presence of bottlenecks was not found. However, in our study, the number of victims in the field hospital is limited, and further evaluations of bottlenecks in simulations with a greater number of victims are necessary.

Similar presence patterns of victims coded with the same triage were observed in the rooms of the field hospital. In particular, the more serious cases (yellow and red codes) spent more time in the Ambulance triage room and the victims coded green spent more time in the Examination room. Our results showed a correspondence between the triage of the victims and the treatment given to the patients from the point of view of the permanence times in the rooms of the field hospital and the interactions with the caregivers (see Section 6 in the Multimedia Appendix 1). In other words, victims with comparable severity injuries were managed in a similar way in the field hospital. The red victim 172 was an exception in this trend: the ideal triage code of the victim 172 was yellow. This result is consistent with the high degree of overtriage of yellow casualties as red occurred in this simulation. It is well known that overtriage may cause fatigue of staff, depletion of resources, and impairment of efficient flow of critically injured patients through the system to definitive care [23]. The use of physical space plays a key role in managing a sudden influx of injured people or patients [24]. The evaluation of casualty flow and hospital space usage during exercises is a necessary first step in disaster preparedness and readiness by hospital authorities.

### Limitations

It is important to highlight some limitations of this study. The exercise was organized as realistically as possible. Despite this, it is still a simulation, and the patients could portray only a limited number of changes in the clinical condition. Another potential issue concerns the possibility that participants changed their behavior because they were wearing sensors and knew they were participating in a scientific measure.

However, the methods presented in this paper can be useful to detect contact patterns in the very specific context of MCIs, thus allowing the implementation of tailored prevention strategies accordingly.

The measurement approach we used here also has limitations. Contacts were defined as face-to-face proximity, but no information on the possible occurrence of a physical contact between the 2 individuals is available [10], and consequently, no information on the interactions of caregiver-casualty is provided. Moreover, the short period of time of data collection (3 hours) also limits the ability to draw conclusions on what happens at longer time scales. However, through the use of the proximity-sensing platform, long-time studies are allowed.

### Conclusions

In conclusion, our study showed that using wearable sensors based on proximity-sensor technology is feasible for obtaining a precise measurement of the pattern of close contacts among individuals during an MCI simulation. Although after-exercise debriefing sessions, during which participants discuss deficiencies warranting improvement, are routinely conducted, there is no commonly used and validated method for evaluating health performance during MCI exercises. Thus, our work constitutes a first step toward a standardized approach to the evaluation of an MCI exercise performance, as this monitoring system provides detailed temporal and spatial information about the medical staff and their interactions with the victims with limited human intervention. It represents, to our knowledge, the first example of unsupervised data collection of face-to-face contacts during an MCI exercise by means of wearable proximity sensors.

The unsupervised measurement of contact patterns with proximity sensors provides a unique opportunity to monitor the interactions between participants without the involvement of direct observers, which could impair the exercise’s realism. Moreover, the analysis of contact patterns may help to identify specific interactions between health staff-patient to evaluate the decisions taken and the performance as the task allocation. In this study, the use of the sensors as fixed devices allowed to analyze the casualty flow in the field hospital to assess the use of physical space and resources allocation. The versatility of the system makes it possible to repeat similar studies in different environments, such as multiple vehicular accident settings or training for terrorist attacks, including smaller settings, and to compare results across contexts. Future studies could include a comparison of contact patterns on different settings of mass casualty simulations to improve the medical process, resource utilization, and decision making.

## Appendix

Multimedia Appendix 1 Additional description of the proximity sensing platform and additional analyses of the caregivers-victims network.

## Abbreviations

DCC: dynamic casualty card
EMDM: European Master in Disaster Medicine
EMS: Emergency Medical Service
HCA: Health Care Assistant
ISS: Injury Severity Score
MCI: mass casualty incident
MD: Medical Doctors
RFID: Radio-Frequency IDentification
t-SNE: t-Stochastic Neighbor Embedding
