# Supplementary material for: Wearable Proximity Sensors for Monitoring a Mass Casualty Incident Exercise: Feasibility Study
Source: J Med Internet Res. 2019 Apr 26;21(4):e12251. doi: 10.2196/12251 (PMC6658323; doi:10.2196/12251)
Supplement: Multimedia Appendix 1 [file jmir_v21i4e12251_app1.pdf]

Multimedia appendix 1

1. Data collection infrastructure

The RFID devices given to the participants exchange ultra-low power radio packets in a peer-to-peer fashion, and perform a scan of their neighbourhood by alternating transmit and receive cycles. During the transmit phase, low-power packets are sent out on a specific radio channel; during the receive phase, the devices listen on the same channel for packets sent by nearby devices. By including the transmitting signal strength in the payload, the receiving device can estimate the degree of proximity of the transmitting device, and in this operation can be carried out in a fully-disseminated fashion throughout the sensing network. Sensors in close proximity exchange with one another a maximum of about 1 power packet per second, and the exchange of low-power radio-packets is used as a proxy for the spatial proximity of the persons wearing the sensors. In particular, close proximity is measured by the attenuation, defined as the difference between the received and transmitted power.

The tags' communication is scheduled via internal clocks that need to be synchronized to a global time reference: before the deployment, 'master' tags receive the time-stamp by connecting the programmer with a PC, and these master tags transmit it to the other tags. The data are stored in the internal memory of sensor, and later retrieved through download from the retrieved tag via the programmer and saved to a PC. After the download, raw data are decrypted, decompressed and saved as binary log files, one for each sensor. Log files can be processed using any programming language and libraries for the analysis of binary data. We performed all our analyses using Python 2.7 and modules of the Python Standard Library along with Numpy (v 1.12) and Pandas (v 0.20).

A time-varying graph based on the data can be generated in the following way. A contact is asserted when the median attenuation of received packets over a 20 second exceeds an attenuation threshold of 70dBm. Changing the attenuation threshold alters the proximity detection range: the higher the attenuation threshold the smaller the detection range.

While the setting described and used for this work permitted to collect data analyzed afterwards, the system also allows live-streaming setting with antennas if wanted. These antennas are connected to a Local Area Network and a central computer collects and stores the information sent by antennas. This information consists in the identity of the badge who has sent the information about its neighborhood, the identity of the badges in this neighborhood, the identity of the antenna and the time at which the information has been sent from the badge to the antenna. Examples of such setting are given in Barrat *et al. 2010.*

2. Triage Accuracy

Here, we confront expected and assigned triage to evaluate the performance of the medical staff during the simulation. Nine victims expected to be dead on the scene were correctly triaged as black at the scene of the accident. Nine expected red casualties evolved negatively on scene and died before transportation and scene evacuation. Triage accuracy (assigned versus expected) was 85% overall. Triage correctness, over and under-triage stratified per severity is presented in Table S1.

Table S1. Correct and incorrect percentage of assigned triage.

		**Incorrect**	
**Triage colour code**	**Correct (%)**	***Over (%)***	***Under (%)***
Red	100	0	0
Yellow	51.86	25.92	22.22
Green	95.35	4.65	0
Black	84.61	0	15.39
**Total**	85.82	8.20	5.98

3. Degree distributions of caregivers

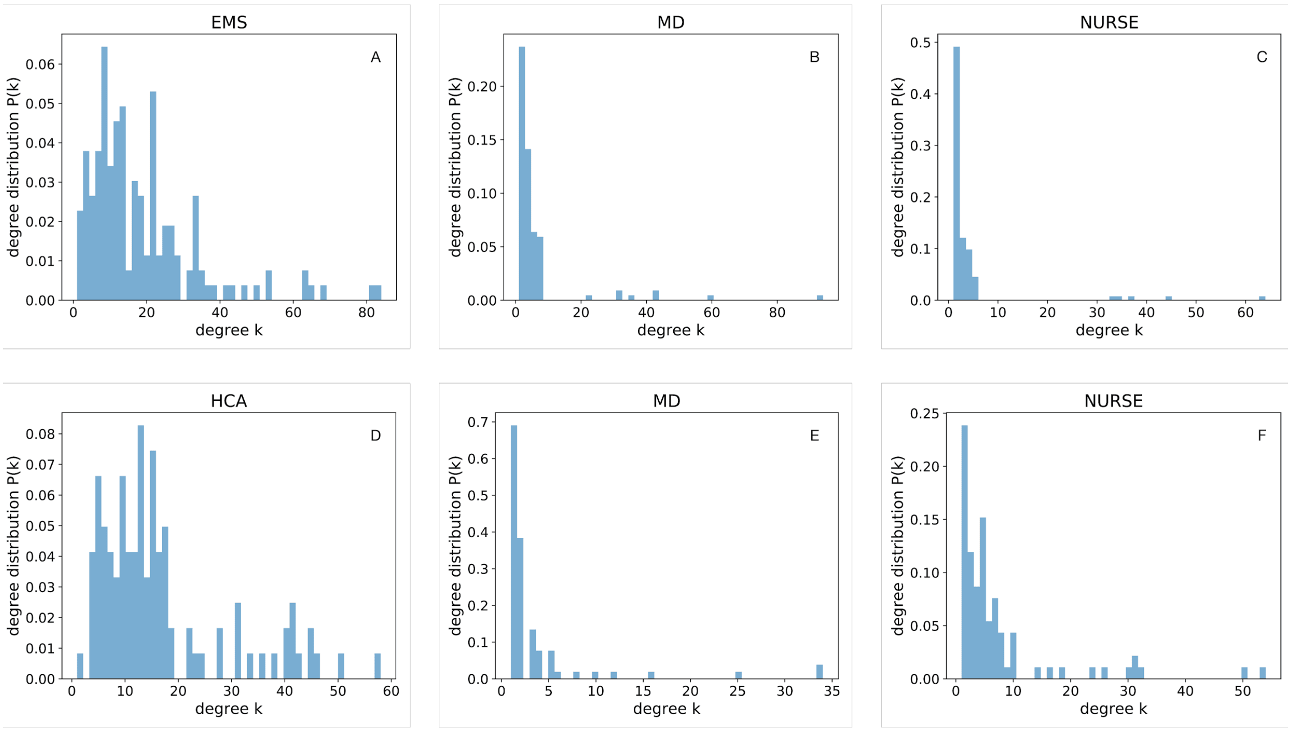

Figure S1. Degree distribution of caregivers: EMS (panel A) on Pre-hospital area; Medical doctors on Pre-hospital area (panel B) and Hospital area (panel E); Nurses on Pre-hospital area (panel C), and Hospital area (panel F); HCA on Hospital area (panel D)

The mean degree of EMS was $\langle k \rangle$ = 18.36, median = 14 (range 1- 84) on Pre-hospital area (panel A). The mean degree of medical doctors was $\langle k \rangle$ = 6.12, median = 3 (range 1 – 94) on scene of the accident (panel B), and $\langle k \rangle$ = 3.52, median = 2 (range 1 -34) on field hospital (panel E). The mean degree of the nurses was $\langle k \rangle$ = 4.08, median = 2 (range 1 – 64) (panel C), and $\langle k \rangle$ = 7.52, median = 5 (range 1 – 54) (panel F) on Pre-hospital and Hospital area respectively. The mean degree of HCA was $\langle k \rangle$ = 16.37, median = 13 (range 1- 58) on Hospital area (panel D).

4. Networks density

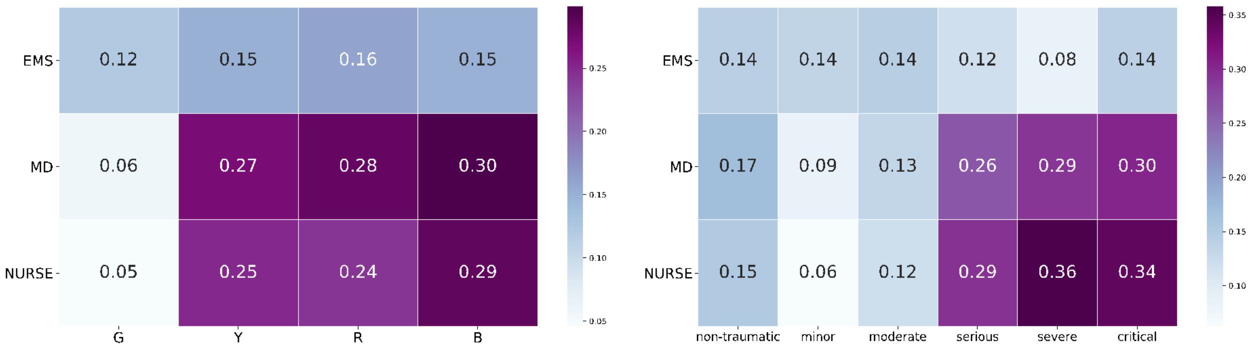

Figure S3. Density of networks in Pre-hospital area. Density of networks (*i.e.*, fraction of all possible edges that are present in the network) built for each caregivers' categories and victims with different triage (right panel), and different ISS (right panel).

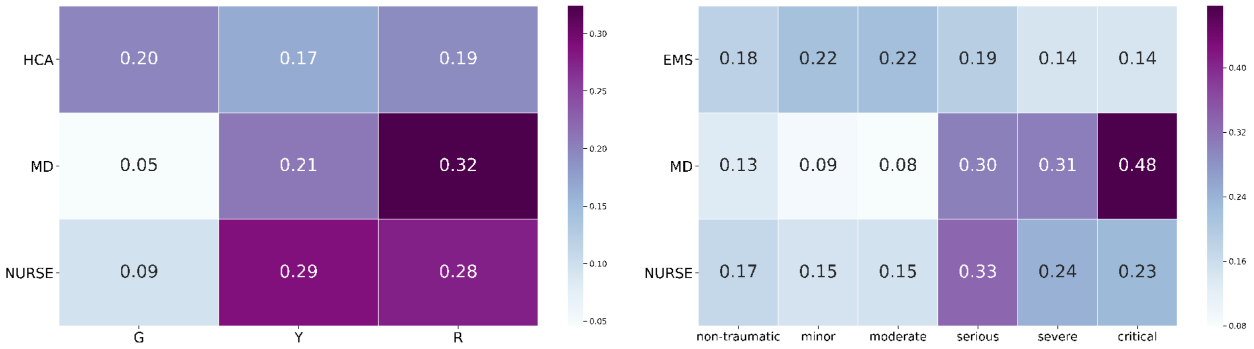

Figure S3. Density of networks in Hospital area. Density of networks (*i.e.*, fraction of all possible edges that are present in the network) built for each caregivers' categories and victims with different triage (right panel), and different ISS (right panel).

5. Contacts temporal evolution

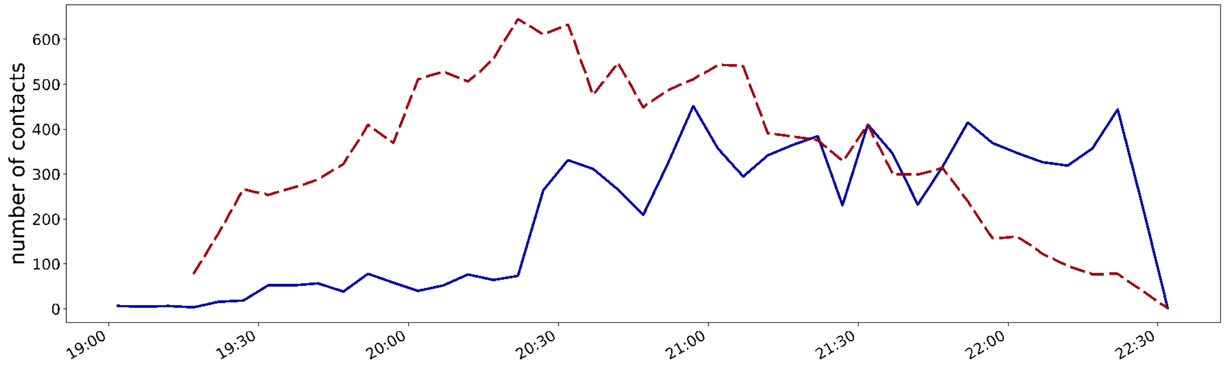

Figure S2. Temporal evolution of the number of contacts on Pre-hospital area (red dashed line) and on Hospital area (blue line). On Pre-hospital area the number of contacts gradually increased until 8:30 PM, and the largest number of contacts occurred between 8:30 PM and 9:05 PM; then the contacts gradually decreased. On Hospital area the number of contacts were stationary until 8:25, then the contacts gradually increased until 10:20 PM. At 8:15 PM patients started to be transferred from Pre-hospital to in-Hospital area.

6. Cumulative time and percentage of time spent in contact

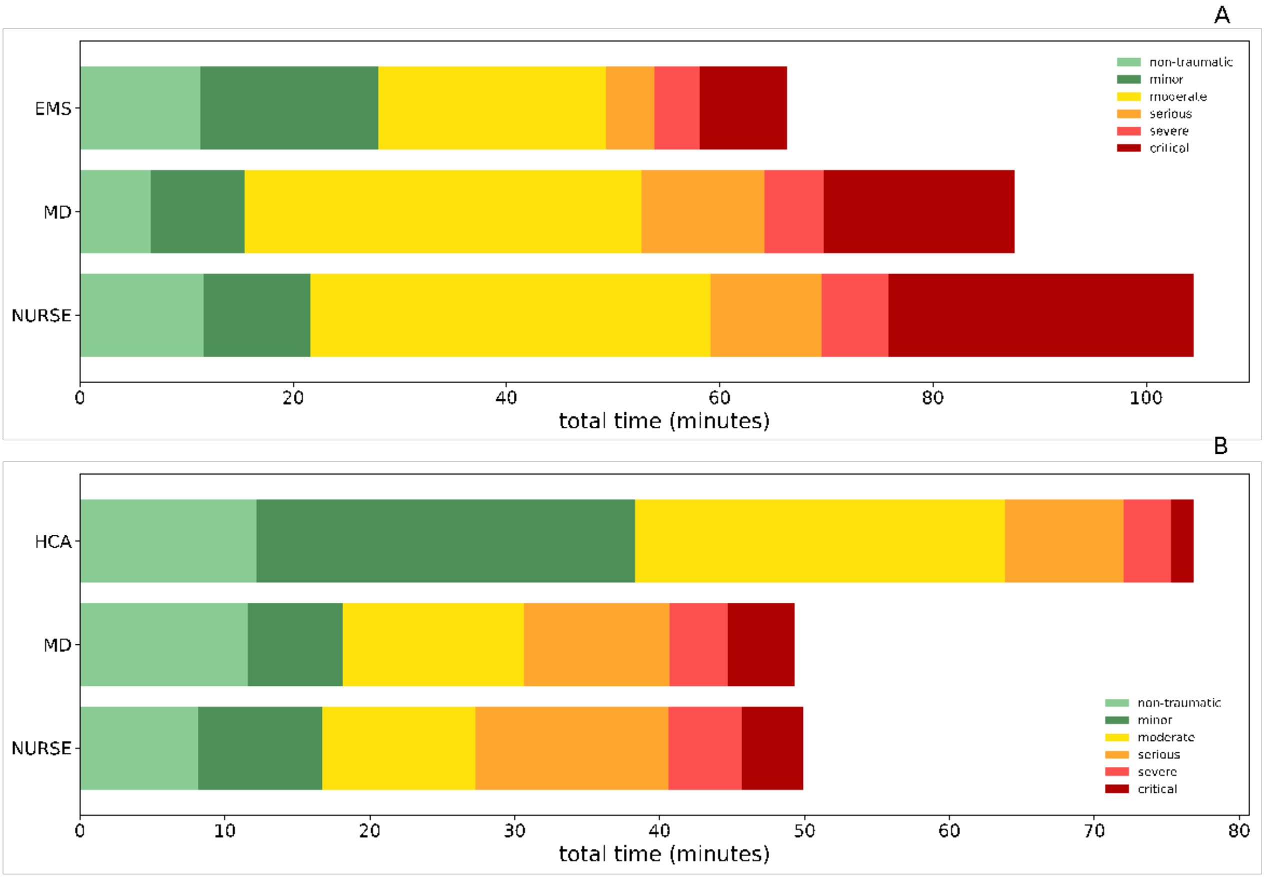

Figure S5. Cumulative time in contact (normalized on total number of participants belonging to each caregiver category) between caregivers and victims with different ISS at Pre-hospital area (panel A) and Hospital area (panel B).

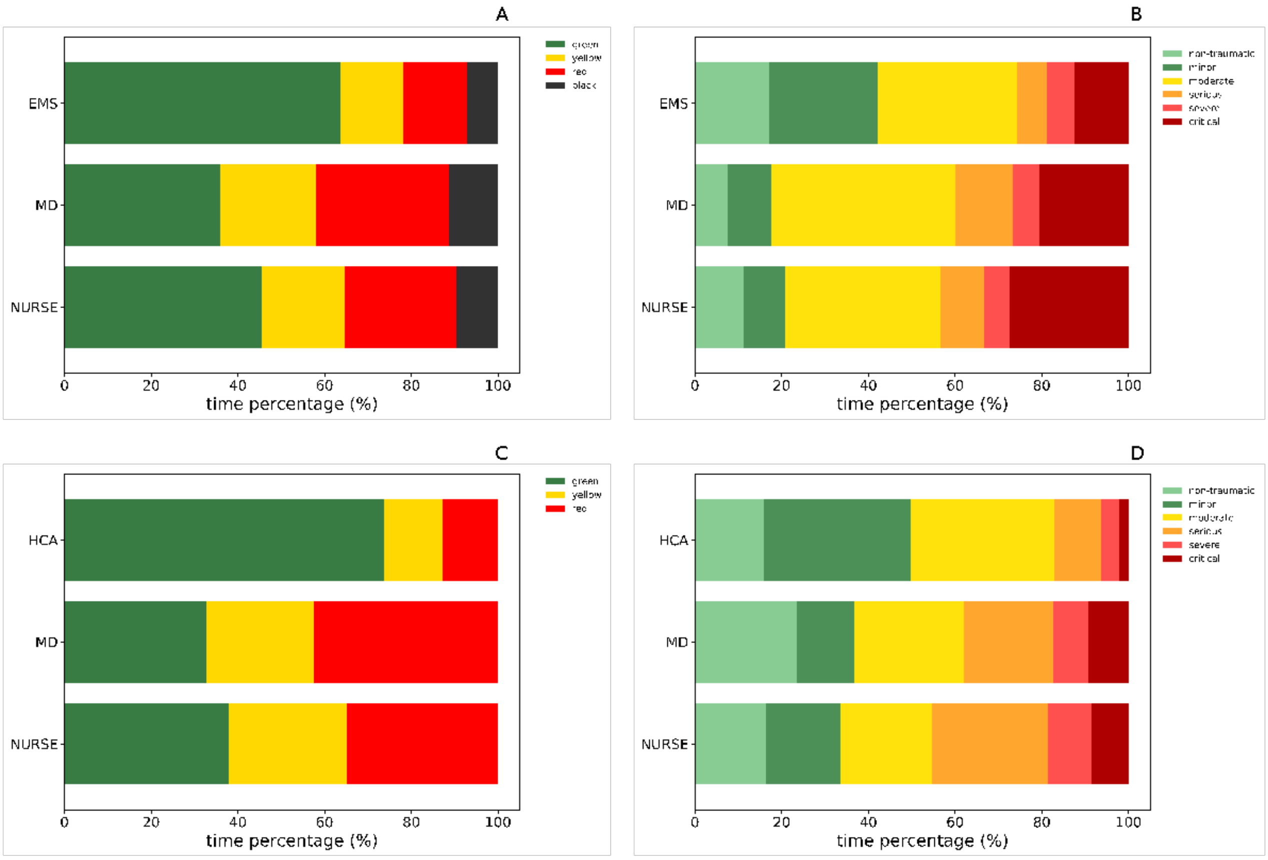

Figure S6. Percentage of time in contact between caregivers and victims with different triage and ISS at Pre-hospital area (panel A, and panel B) and Hospital area (panel C, and panel D).

References

Barrat, A., Cattuto, C., Szomszor, M., Van den Broeck, W., & Alani, H. (2010, November). Social dynamics in conferences: analyses of data from the Live Social Semantics application. In *International Semantic Web Conference* (pp. 17-33). Springer, Berlin, Heidelberg.
